# The Association between Environmental Factors and Scarlet Fever Incidence in Beijing Region: Using GIS and Spatial Regression Models

**DOI:** 10.3390/ijerph13111083

**Published:** 2016-11-04

**Authors:** Gehendra Mahara, Chao Wang, Kun Yang, Sipeng Chen, Jin Guo, Qi Gao, Wei Wang, Quanyi Wang, Xiuhua Guo

**Affiliations:** 1 Department of Epidemiology and Biostatistics, School of Public Health, Capital Medical University, Beijing 100069, China; gbmahara@163.com (G.M.); yangkun_1123@163.com (K.Y.); ivanchen2010sp@hotmail.com (S.C.); guojin5827501@163.com (J.G.); gaoqi@ccmu.edu.cn (Q.G.); wei.wang@ecu.edu.au (W.W.); 2 Beijing Municipal Key Laboratory of Clinical Epidemiology, Beijing 100069, China; 3 Department of Statistics and Information, Beijing Center for Disease Control and Prevention (CDC), Beijing Center for Preventive Medical Research, Beijing 100013, China; 13810147054@139.com; 4 Systems and Intervention Research Centre for Health, School of Medical Sciences, Edith Cowan University, Perth 6027, Australia; 5 Institute for Infectious Disease & Endemic Disease Control, Beijing Center for Disease Control and Prevention (CDC), Beijing Center for Preventive Medical Research, Beijing 100013, China

**Keywords:** spatial regression analysis, scarlet fever, meteorological factors, air pollutant factors, Beijing

## Abstract

(1) Background: Evidence regarding scarlet fever and its relationship with meteorological, including air pollution factors, is not very available. This study aimed to examine the relationship between ambient air pollutants and meteorological factors with scarlet fever occurrence in Beijing, China. (2) Methods: A retrospective ecological study was carried out to distinguish the epidemic characteristics of scarlet fever incidence in Beijing districts from 2013 to 2014. Daily incidence and corresponding air pollutant and meteorological data were used to develop the model. Global Moran’s *I* statistic and Anselin’s local Moran’s *I* (LISA) were applied to detect the spatial autocorrelation (spatial dependency) and clusters of scarlet fever incidence. The spatial lag model (SLM) and spatial error model (SEM) including ordinary least squares (OLS) models were then applied to probe the association between scarlet fever incidence and meteorological including air pollution factors. (3) Results: Among the 5491 cases, more than half (62%) were male, and more than one-third (37.8%) were female, with the annual average incidence rate 14.64 per 100,000 population. Spatial autocorrelation analysis exhibited the existence of spatial dependence; therefore, we applied spatial regression models. After comparing the values of R-square, log-likelihood and the Akaike information criterion (AIC) among the three models, the OLS model (R^2^ = 0.0741, log likelihood = −1819.69, AIC = 3665.38), SLM (R^2^ = 0.0786, log likelihood = −1819.04, AIC = 3665.08) and SEM (R^2^ = 0.0743, log likelihood = −1819.67, AIC = 3665.36), identified that the spatial lag model (SLM) was best for model fit for the regression model. There was a positive significant association between nitrogen oxide (*p* = 0.027), rainfall (*p* = 0.036) and sunshine hour (*p* = 0.048), while the relative humidity (*p* = 0.034) had an adverse association with scarlet fever incidence in SLM. (4) Conclusions: Our findings indicated that meteorological, as well as air pollutant factors may increase the incidence of scarlet fever; these findings may help to guide scarlet fever control programs and targeting the intervention.

## 1. Introduction

It has been well known that scarlet fever is a seasonal infectious childhood illness caused by Group A *Streptococcus* bacteria (Group A beta haemolytic streptococci (GABHS)). Scarlet fever predominately occurs in school children aged between 2 and 10 years. Generally, it spreads by the aerosol route (inhalation), but may also be able to spread through skin contact or by fomites [1,2,3,4]. So far, it is not normally considered as a food- or milk-borne illness, as an outbreak noted in China due to infected chicken meat [5,6]. There are currently no vaccines available to protect against *S. pyogenes* infection (the vaccine developed by George and Gladys Dick in 1924 was discontinued due to its poor efficacy) [2,3]. However, this disease can be fully cured and complications prevented by antibiotic application [1,2,3]. Remarkably, the trend of scarlet fever outbreaks has been increasing among the school children in mainland China and other countries, as well [7,8,9,10]. Therefore, it is still regarded as an important public health problem in China [11].

Several studies have been conducted previously to evaluate the association between meteorological factors and scarlet fever incidence; however, the findings have been varied. Temperature, wind speed and sunshine time have an established positive correlation with scarlet fever [12,13,14], while average temperature and sunshine time have a negative correlation with scarlet fever incidence revealed in a few studies [12,14]. Similarly, a study showed that scarlet fever incidence was negatively correlated with temperature and relative humidity, while it was positively associated with the mean temperature [15]. Likewise, a study provided evidence that the incidence of scarlet fever with monthly average pressure, monthly lowest temperature and monthly relative humidity were the factors that influence the disease [16].

Most of the previous studies considered the methodology, such as multiple linear regression models, negative binomial regression, the generalized additive Poisson model (GAPM), fast Fourier transformation (FFT), simple or uni-factorial correlation and multiple stepwise regression, and rarely focus on spatial and time dimension (time series analysis) approaches, which might cause the loss of information by ignoring the heterogeneity in both time and space and result in different conclusions [12,13,14,15,16,17,18,19,20,21,22]. The majority of the above studies have been conducted using simple descriptive statistics method and published in Chinese language journals. Understanding such spatial variations in scarlet fever incidence and its determinant factors within a social, environmental and temporal context are essential for improved targeting of an intervention and resource allocation. However, very few studies applied the geospatial analytical methods (GIS) to investigate the epidemic characteristic of scarlet fever. It is difficult to incorporate the spatial heterogeneity of the effects of ecological factors (meteorological and air pollutant factors) with those traditional methods [23,24]. In this regard, the geographic information system (GIS) is the preferred method and technique for the underlying epidemic characteristics of an infectious disease [25], which allow one to attain more easily and more efficiently data visualization and manipulation, as well as to analyse and display the geographic data [26,27,28]. However, such studies have not been conducted yet to investigate the geospatial distribution of scarlet fever in the Beijing region using GIS technology. Spatial regression models are more explanatory methods compared to the traditional regression methods on the basis of cross-sectional or time series data alone, which can enable the researcher to control for both spatial dependency and unknown heterogeneity [29]. In fact, spatial regression methods capture spatial dependence in regression analysis and avoid statistical problems for, e.g., unstable parameters and unreliable significance tests along with providing the information on the spatial relationship among the variables [30]. Moreover, we could not find such a study to date similar to our objectives, for example the association between ambient air pollutant variables (PM_2.5_, PM_10_, NO_2_, SO_2_, O_3_, CO) along with meteorological variables and scarlet fever incidence in the study settings.

Therefore, we performed spatial regression models to explore the association between environmental factors (air pollutant variables, meteorological variables) with scarlet fever incidence; because of recent years, air pollution is a serious public health issue in China, especially in megacities like Beijing [31,32].

## 2. Methods

### 2.1. Study Area and Design

A retrospective ecological study was conducted in Beijing region at the district level based on the reported cases of scarlet fever from 2013 to 2014. Beijing is the capital of the People’s Republic of China, situated at the northern tip of the North China Plain, at 39°56’ N and 116°20′ E, and surrounded by mountains to the north, northwest and west. The topography of the Beijing area slopes from the northwest to the southeast, and there is a continental climate characterized by hot, humid summers and cold, windy and dry winters. The total area of the Beijing region is 16,410.54 km^2^, with a population of 21.15 million, divided into 16 administrative districts [33].

### 2.2. Data Source of Scarlet Fever

Scarlet fever cases were the outcome variable. The daily reported cases of scarlet fever data of each district in Beijing from January 2013 to November 2014 were obtained from the Center for Disease Prevention and Control China (CDC). All cases were diagnosed according to the clinical criteria established by the Law of Communicable Diseases Prevention and Control of the People’s Republic China, a guidebook published by the National Health and Family Planning Commission of the People’s Republic of China. Scarlet fever is a notifiable Group B infectious disease, according to the China National Notifiable Infectious Disease Surveillance System (NNIDSS) [7]. Socio-demographic data for each district were obtained from the Beijing Bureau of Statistics of China [34]. The case definition has been published in our earlier publication of Mahara et al. [35].

### 2.3. Ethical Approval

The ethical approval and the consent of each individual subject were not required because we used only aggregated data from CDC, China (the number of cases for each county in months). 

### 2.4. Air Pollutants and Meteorological Data

District-/county-level daily recorded climate factors data of Beijing region from January 2013 to November 2014 were obtained from the China Meteorological Data Sharing Service System [36]. For the further analysis, monthly meteorological variables, such as average atmospheric pressure (AAP), average temperature (AT), average relative humidity (ARH), monthly rainfall (MRF), sunshine hour (ASH) and average wind speed (AWS), were included.

Likewise, daily-24 h real-time air pollutant data, such as concentrated data of PM_2.5_ (µg/m^3^), PM_10_ (µg/m^3^), sulphur dioxide (SO_2_) (µg/m^3^), ozone (O_3_) (µg/m^3^), nitrogen dioxide (NO_2_) (µg/m^3^ unit) and carbon monoxide (CO) (mg/m^3^), were obtained from the Beijing Environmental Protection Bureau (there are 35 ambient air quality monitoring stations in 16 districts in Beijing city) during the study period [37]. Then, we used monthly average data of all environmental factors for the further analysis.

### 2.5. Statistical Analysis

#### 2.5.1. Spatial Analysis

In recent years, spatial analysis has been broadly implicated to describe the geographic distribution of disease patterns and to identify the factors associated with disease incidences [18,38,39,40,41]. It could adjust the mutual effect among the spatial neighbouring features, which may be ignored by traditional regression analysis [42]. In our study, spatial autocorrelation and regression analysis were conducted to detect the disease aggregation and relationship between disease and environmental factors. In the spatial analysis, 16 districts of Beijing area were included. The monthly average incidence of scarlet fever including other environmental variables from January 2013 to November 2014 was included. All meteorological and air pollutant variables were included in the shapefile of the Beijing map according to the Beijing district ID using ArcGIS software Version 10.0 (ESRI Inc., Redlands, CA, USA). The district–level polygon map of Beijing area was gained from the Data Sharing Infrastructure of Earth System Science (data sharing infrastructure base map of Beijing) [43].

#### 2.5.2. Global Spatial Autocorrelation Analysis

In order to provide the information about the presence of the spatial dependency of the dependent variable, the global Moran’s *I* and Anselin’s local Moran’s *I* were correspondingly used for each year in the study area. Spatial autocorrelation measures whether the disease patterns are clustered/dispersed or randomized [44,45,46]. To assess the significance of the Moran’s *I* against a null hypothesis of spatial autocorrelation, we used permutation procedure (randomization) at 999 on Open GeoDa free software to select the suitable significant value. A statistically-significant estimation of Moran’s *I* (z-score ≥ 1.96) indicates that the neighbouring districts have a similar incidence of scarlet fever clusters [45,46,47,48]. All spatial statistics were estimated after log transformation of the dependent variable on SPSS V-20 (SPSS Statistics 20, IBM: Corporation 1 New Orchard Road, Armonk, NY, USA) to meet the criteria of spatial analysis, because the incidences of scarlet fever disease were not normally distributed and highly skewed; thus, incidences of diseases were log transformed. The Open GeoDa environment 1.8.6 (Luc Anselin, Phonix, AZ, USA) and ArcGIS 10.1 (ESRI Inc., Redlands, CA, USA) software were used to perform the above analysis [49].

#### 2.5.3. Spatial Regression Analysis

To explore the relationship between scarlet fever incidence and environmental factors, we applied spatial regression analysis. Traditional regression models assume that the observation is mutually independent, which is not valid due to the spatial structure of data; therefore, random effects are involved to explain the potential impact caused by the spatial correlation [50]. The spatial regression model is expressed as follows:

The study assumed that scarlet fever has several influencing factors to be an infection, such as: air pollutant factors (PM_2.5_, PM_10_, O_3_, NO_2_, SO_2_, CO) and meteorological factors (temperature, relative humidity, air pressure, speed of the wind, rainfall and sunshine time). Therefore, we annualized average values that were used to explore the predictors of scarlet fever incidence, increase the stability of data and minimize the potential bias [45]. The average incidences of scarlet fever at districts level over the 2-year study period were also calculated.

Then, we constructed the shapefile of the dependent variable (outcome) and independent (predictors) variables in ArcGIS software and exported it into the GeoDa environment software for advanced geospatial analysis. GeoDa software is the newest spatial data analysis tool developed by the Center for Spatially Integrated Social Sciences (CSISS) to apply various exploratory spatial data analysis, including data manipulation, mapping and spatial regression analysis [51]. We generated a spatial weights matrix, which is necessary for the calculation of spatial autocorrelation statistics [51]. Spatial weights can be constructed in two ways; either based on contiguity from polygon map files or based on the distance between points. We selected contiguity based on spatial weights, since our main interest is to know the spatial interdependence between the outcome variable and a set of exposure variables in the neighbouring districts. GeoDa software further offers two types of spatially-contiguous weights; these are the rook’s weight (uses common boundaries to define the neighbour) and the queen’s weight (including all common points that are boundaries and vertices). Finally, we used the rook’s contiguity (Figure S1 in Supplementary Materials) weight for estimating all geospatial statistics and spatial regression analysis [51].

Firstly, we used the classical ordinary least square (OLS) model to estimate the effect of various environmental factors on the outcome variable. After knowing the presence of spatial dependence in the outcome and predictor variables, we understood that the assumption of independent observations and errors of classical statistical models might be violated. Therefore, we estimated and compared three regression models, ordinary least square (OLS), the spatial lag model (SLM) [52] and the spatial error model (SEM) [53], to inspect the relationship between the outcome variable with a set of predictors.

Spatial regression methods estimate spatial dependency in regression analysis, avoiding statistical problems, such as unstable parameters and unreliable significance tests, and provide information on spatial relationships among the parameters involved in the model [52,53,54].

The OLS regression model takes the following form:

(1) y=∝+βx+ε

the spatial lag model (spatial auto-regressive model) form:

(2) y=∝+ρWγ+βx+ε

and then, the spatial error model form:

(3) y=∝+ρWγ+βx+ε, with ε=λWε+ζ

where y denotes the scarlet fever incidence, ∝ is an intercept, β is the vector of regression parameters, x is the matrix of exogenous explanatory variables, ԑ is the vector of the random error term, Wγ is the spatial lag term, ρ is the spatial autoregressive parameter of Wγ (which is estimated for the model as a whole) and λ is the coefficient of spatially-lagged autoregressive errors, Wε. Errors in ζ are independently distributed, and *W* is the spatial weight [52,53,54].

In the spatial analysis, a key step is to determine whether the spatial lag model or spatial error model is more applicable for a given process. For this, a very popular method is the LM (Lagrange multiplier) test in empirical research [52,53,54]. Four Lagrange Multiplier test statistics were used in this analysis as the diagnostics output; LM lag, LM error, robust LM lag and robust LM error. These diagnostics test results can be obtained from the OLS model and spatial weights matrix. The LM error or LM lag statistics are used to test whether the spatial error models or spatial lag models are the appropriate alternatives. LM lag and robust LM lag tests relate to the spatial lag model as appropriate regression models, while both the LM error and lag statistics are not significant (i.e., do not reject the null hypothesis, no spatial dependence); the analysis should use the OLS results only. Likewise, if the LM error test rejects the null hypothesis, but LM lag does not, then select the spatial error model in the regression process, and vice versa. If both LM error and lag statistics reject the null hypothesis, the robust LM test can be applied. When both robust LM error and lag test statistics are highly significant, the analysis may select the model with the larger value of the statistics [52,53,54,55].

It should be underlined that the choice of spatial lag or error models should not be based on statistical analysis, and it is actual previous theoretical knowledge [54,55]. If the omitted parameters are expected in a study due to unobservable spatially-correlated factors, then SEM models may be suitable. In contract, if the analyst wants to emphasize the neighbourhood effects, the SLM models may be better than the SEM model [52,53].

Three regression models (classical ordinary least squares (OLS) regression models, global spatial regression models), the spatial lag model (SLM) and the spatial errors model (SEM) were effectively applied to explore the relationship between environmental factors and scarlet fever incidence in the study region. The OLS model was first performed on each explanatory variable with the dependent variable and a consistently performed spatial dependence test on the SLM and SEM model, then comparing these three types of models with the findings of R-square, log likelihood ratio and AIC were used to decide which model is appropriate for the goodness of model fit [52,55]. 

Software SPSS 20.0, ArcGIS 10.2.2 (ESRI Inc., Redlands, CA, USA) and GeoDa were used for data analysis. The country boundary electronic map of Beijing region was intercepted from the Data Sharing Infrastructure of Earth System Science (data sharing infrastructure base map of Beijing [44]).

## 3. Results

### 3.1. Basic Characteristics

A total of 5491 scarlet fever cases was reported in 16 districts in Beijing from January 2013 to November 2014. The annual average incidence rate of scarlet fever was 14.63 per 100,000 population, and the total numbers of cases were 2170 in 2013 and 3321 cases in 2014. Among the total cases, 62.1% (3414) were male and 37.8% (2017) female. Most of the scarlet fever patients were 3–8-year-old children (83%) followed by 9–15 years (14%), and the other age group accounted for only 3% during the study period.

Table 1 shows the descriptive information of the outcome and explanatory variables. The overall monthly mean PM_2.5_ concentration was 91.71 (range of 41.80 to 269.40 µg/m^3^) during the study period. According to the Chinese Ambient Air Quality Standards, it is the Grade II standard for 24-h average PM_2.5_ concentrations (real-time air quality index (AQI ≤ 75 µg/m^3^). Similarly, the average monthly mean concentrations of PM_10_ (121.77 µg/m^3^, SO_2_ (28.06 µg/m^3^), NO_2_ (52.75 µg/m^3^), O_3_ (117.40 µg/m^3^) and CO (1.73 µg/m^3^) including meteorological variables; rainfall (1.46 inches), air pressure (992.11 hPa), temperature (12.26 °C), relative humidity (57.06%), wind speed (2.10 km/h) and sunshine hour (6.54 h) were recorded, which are listed in Table 1. 

**Figure 1:**
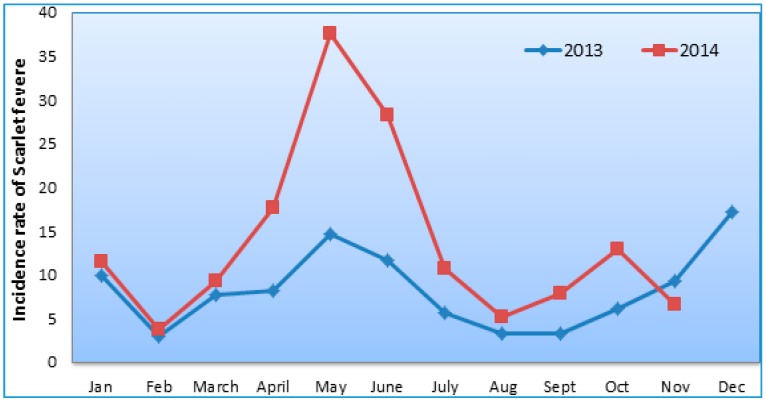
Scarlet fever incidence in Beijing districts, 2013 to 2014.

**Figure 2:**
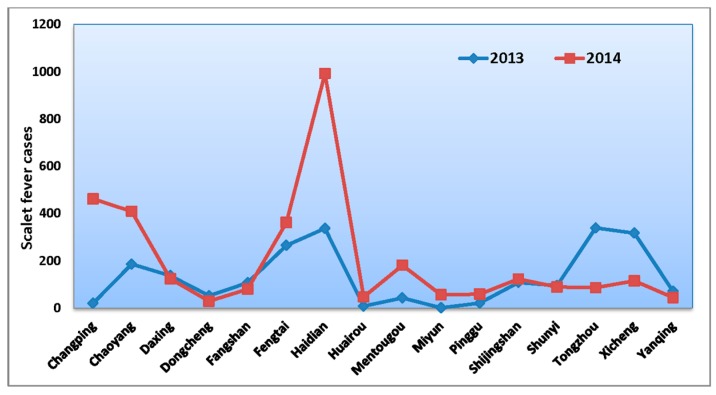
Distribution of scarlet fever cases in Beijing districts, 2013–2014.

Figure 1 exhibits the monthly trends of the incidence rate of scarlet fever, which had a seasonality and periodicity in the Beijing region during the whole study period (Figure 1 and Figure 2). The first large seasonal peak occurred in May (between March and July), followed by a small peak in December (from October to January). 

### 3.2. Spatial Autocorrelation of Scarlet Fever Incidence

**Figure 3:**
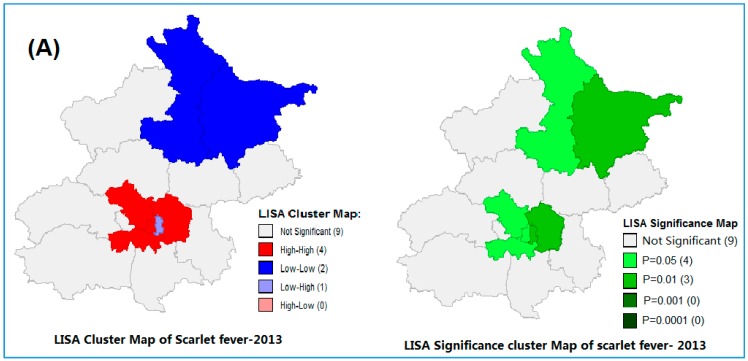

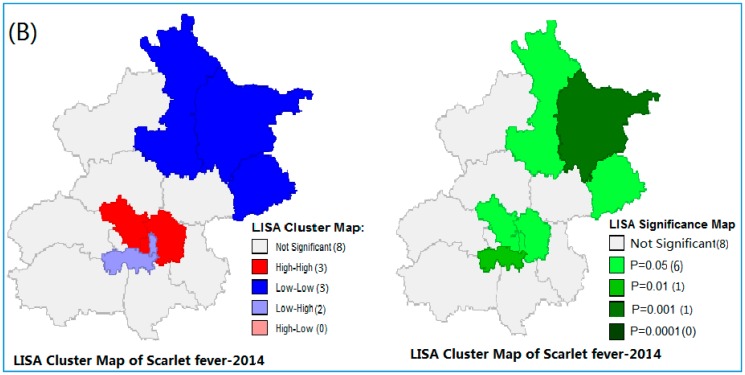
Local Moran’s *I* analysis (LISA) of Scarlet fever clusters in Beijing, from (**A**) 2013 and (**B**) 2014.

The global spatial autocorrelation analysis of scarlet fever for each year is shown in Figure S2 (in Supplementary Materials), which demonstrated a significant spatial autocorrelation of scarlet fever at the district level in Beijing during the study period (2013–2014). The high Moran’s *I* values were from 0.12 to 0.13 (*p-*value range from 0.015 to 0.038). We performed local Moran’s *I* statistics to explore the clustering of disease incidence in the study region (Figure 3A,B). Anselin’s local Moran’s showed the core clustering of high-high, low-high and low-low of scarlet fever incidence districts, which indicates the existence of spatial dependency on the occurrence of scarlet fever (Figure 3A,B). Therefore, we performed spatial regression analysis to estimate the association between scarlet fever and ecological, as well as air pollutant variables. 

### 3.3. Spatial Regression Analysis

Statistical test results of the classical ordinary least squares (OLS) model estimation were used to determine in which specific fixed effect and what type of spatial dependence term should be included in the model (Table S1 in Supplementary Materials). The low probability (*p* < 0.001) of the test score indicated the non-normal distribution of the error term. The low probability of the three test points presents heteroscedasticity. In our study, all simple tests of the LM lag, LM error, robust LM lag and robust error and Lag multiplier (SHARMA) test are significant at (*p* < 0.05), indicating the presence of spatial dependence (Table S1 in Supplementary Materials). We employed the OLS model to estimate the effect of various predictors on the outcome variable. Our result suggested that PM_2.5_, PM_10_, SO_2_, O_3_, NO_2_, MRF, AAP and ASH were positively associated with scarlet fever, while CO, AT, ARH and AWS were negatively associated with scarlet fever in the OLS model (Table 2).

After identifying the presence of spatial dependence in the OLS model, we re-estimated the regression models (SLM and SEM) with the maximum likelihood approach while controlling for the spatial dependence. We performed the spatial lag term of scarlet fever with the designed spatial weight file. The coefficient parameter (Rho) reveals the spatial dependence inherent in our sample data that measuring the average influences the observations by their neighbouring observations. It has a positive effect, and the general model fit improved compared to OLS model, which indicated higher values of R-square and low values of the log likelihood and AIC. The effects of other independent variables remain nearly the same. So far, we conclude that the introduction of the spatial lag term improved the model fit (Table 2).

After comparing with the spatial lag model output, we also designed a spatial weight file and a coefficient on the spatially-correlated error (LAMBDA), which is added as an additional indicator. It has a positive effect that is highly significant. Therefore, allowing the error terms to be spatially correlated improved the model fit, such that we could not make the spatial effects go away.

By comparing the model parameter values of R-square, log-likelihood and AIC, we found that the spatial lag model and spatial error models were better fit than the classical fixed effects (OLS) model, and that the spatial lag model has greater R-square, log likelihood and a low AIC value compared to the spatial error model, which provides a statistical basis to adopt this model for the goodness of model fit. After considering the spatial coefficients of both the spatial lag and spatial error models, it was indicated that the scarlet fever incidence of a spatial unit correlates with the surrounding spatial units.

Average monthly nitrogen dioxide (NO_2_), average monthly rainfall (ARF) and average sunshine hour (ASH) had a significant positive correlation with scarlet fever incidence, while average relative humidity (ARH) has a significantly negative correlation with scarlet fever incidence during the study period (Table 2). Similarly, there was a positive correlation coefficient found between PM_2.5_, PM_10_, SO_2_, O_3_ and APP with scarlet fever incidence, whereas CO, AT and AWS had a negative relationship established with scarlet fever incidence; however, these variables were not statistically significant in SLM (Table 2).

## 4. Discussion

This study, for the first time, investigated the association between scarlet fever with meteorological factors along with air pollutant variables using spatial regression models based on a longitudinal data in 16 districts/counties in the Beijing region from January 2013 to November 2014. Our study detected that the annual incidence of scarlet fever was predominantly higher among boys compared to girls, particularly in the nursery, kindergarten and pre-primary school children during our study period. Consistent findings were revealed by several studies in different regions [1,9,10,11,54,55,56,57,58].

The occurrence of the epidemic form of scarlet fever is to be determined by a several factors, e.g., environmental conditions, the nature and prevalence of the microorganism and some other distribution factors and resistance of the host-population. Some of these components are measurable, and others are not. Of the environmental factors, climate and seasons are both important, for scarlet fever disease is known in the tropics, and its incidence has a rough relation to the geographical location of that particular country. Seasonally, scarlet fever incidence in the Beijing area showed that there was a large seasonal peak between March and July, followed by a small peak in October to January, which was similar to other studies [1,9,54,55,56,57,58]. This seasonal increase has no relation with the occurrence of respiratory catarrhal infection, but seems to be closely related with the reassembly of schools. Evidently, the seasonal peaks in different regions might be varied according to the geographic variation along with environmental factors [17]; for example, the United States scarlet fever cases generally peak in the winter months [11]. In the Beijing region, the scarlet fever incidence patterns were exhibited with a high Moran’s *I* value from 0.12 to 0.13 (*p* = 0.015–0.038) over the study period. Likewise, local Moran’s *I* findings demonstrated that the incidence cluster districts have changed over time in the study area. It has clearly witnessed that high-high disease incidence cluster districts were reduced in 2014 compared to 2013, that is four cluster districts to three cluster districts, which is a significant change over time, and it should be a great achievement in those places where population density is high. Population density and overcrowded situations are also associated risk factors for scarlet fever incidence, which has been established from our earlier study [35]. This finding is consistent with the study of Qian Hi-Kun et al. in Beijing [18].

Our study found a positive significant relationship between average monthly rainfall (5.498, *p* = 0.036) and average sunshine hours (3.8136, *p* = 0.048) with scarlet fever incidence, which was consistent with the findings of other studies [12,13,15]. Contrasting this, mean temperature, monthly minimum temperature and sunshine hour had a negative correlation with scarlet fever incidence revealed in some studies [12,14,15]. Our study also established that an average relative humidity (−0.6816, *p* = 0.034) was significantly negatively correlated with scarlet fever incidence, which was a similar finding to the recent study of Duan Yu et al. [15] in Hefei city, China. Previous studies have revealed that there was a positive association between average air pressure and wind speed with scarlet fever incidence in Beijing city and Hefei city in China [13,14,15,16]. However, our study established a negative association between scarlet fever and average wind speed (−9.69804, *p* = 0.122), which was not statistically significant during the study period. This may be due to the study period, because most of the previous studies had been done by using the dataset before 2010 [13,14,15,16]. Additionally, a previous outbreak investigation of streptococcal pyogen in military training centres established that environmental contamination with *Streptococcus pyogenes* was common, and contact with contaminated substances contributed to the transmission of the bacterium [59]. To reduce this transmission, disinfection measures can be applied. If we observe the past relationship between rainfall and scarlet fever prevalence in the 18th century (1855 to 1875) throughout Europe, Great Britain and the U.S., even though a positive relationship appeared, that means wet years being accompanied had low and dry years had more prevalence of scarlet fever [60]. This dry condition (spring/summer) has a positive relationship with scarlet fever in England and Wales in the 19th century [61], and consistently, a positive correlation has been appearing until recent years [1,10,55,56,57,58,59]. The explanation behind this could be that *Streptococcus pyogenes* may survive better and be more active in dry weather than wet conditions.

Additionally, there are several pieces of evidence that environmental factors, such as air pollutant factors (PM_2.5_, PM_10_, O_3_, NO_2_, SO_2_, CO), may influence the disease occurrence in society [31,32]. In this regard, due to having high ambient air pollution in the provincial capitals in China, including Beijing city [31,32,62,63], our study first attempted to explore the relationship between scarlet fever incidence and air pollutant factors based on reported cases of scarlet fever. Our study observed that NO_2_ (0.35493, *p* = 0.027) was significantly associated with scarlet fever incidence in the study setting. However, we could not find any previous evidence regarding this. Moreover, current scientific evidence suggested that short-term NO_2_ exposures ranging from 30 min to 24 h are linked to adverse respiratory effects, including airway inflammation in healthy people and increasing respiratory symptoms [64]. Based on our knowledge, scarlet fever also affects the upper respiratory track, which may support the sore throat mechanism. Therefore, high NO_2_ exposure is more likely to increase scarlet fever, as well. However, no studies have yet been published revealing the underlying mechanism; further studies should be done to explore these things more precisely. Other remaining air pollutant factors were not statistically significant in our study, and earlier, we did not find any scientific evidence between scarlet fever and the above factors. From these findings, we can assume that there is no direct relationship with bacterial infections with air pollutant factors. To understand more details regarding these issues, further studies are needed in the region.

In addition to the disease occurrence, there should be biological agents present along with other supporting factors, as we mentioned previously. Group A *Streptococcus pyogenes* (GAS) is a major human causative organism of a wide spectrum of clinical diseases, including scarlet fever [1]. The stain characteristics of GAS and its specific virulence factors have been measured to explain the frequency and severity of GAS disease [4]. Among the 74 species under the genus of *Streptococcus*, *Streptococcus pyogenes* is one of the most virulent species causing human infections [11]. It is a prototype bacterium with M encoded by *emm* typed, which is considered to be a major factor for disease transmission [11]. The *emm12* and *emm1* type of gene have commonly been isolated in mainland China during scarlet fever epidemics [54,57]. In contrast, *emm1* (39%), *emm 12* (13%), *emm89* (9%) and *emm28* (4%) typed *emm* were recorded in the United Kingdom recently [65]. Isolated scarlet fever cases immediately after the outbreak was noted are used to identify the genus of the biological agent for better treatment and reduce the complications. On the other hand, it is predicted that *emm12* typed *Streptococcus pyogenes* with resistance to tetracycline and macrolide antibiotic drugs was the cause of scarlet fever epidemics in past years in Hong Kong and mainland China [65]. Thus, drug resistance is also one associated factor for scarlet fever. However, our research could not include this factor in our study, due to the ecological nature of the study.

Although this study appraised the association between scarlet fever with environmental factors by using spatial regression models, there are some limitations that should be acknowledged. First is that the effects of many factors, for example social and economic status, existing health services and hygiene, were not quantified precisely due to unavailable data at the district level. Second, we assumed that the effect of meteorological variables on scarlet fever was consistent for all study areas. However, it would be difficult to keep the spatial stationary assumption in a large area. Thus, the geographically-weighted regression model (GWR) would be used to analyse the local effect of factors of interest in the future [30]. Third, we selected a month as a temporal scale for more accurate outcomes or lag effects of meteorological and air pollutants factors, which might have been accomplished if detailed data were obtained and used for the analysis. Fourth, the data were obtained from an official surveillance system, which cannot omit the possibility of the underreporting of cases. Cases may be missed by the routine notification system. Last, this is an ecological study, which is exploring the association at the group level, so ecologic fallacy is the critical limitation of this study, and the best way to reduce ecologic fallacy is multi-level modelling, which includes both individual and ecological level factors [66].

## 5. Conclusions

Our study provided comprehensive evidence that the incidence of scarlet fever could be affected by meteorological, as well as air pollution factors. The exploration of spatial regression predictors identified nitrogen oxide, rainfall and sunshine hours had a positive association, while relative humidity had a negative association with scarlet fever incidence. Our findings may provide assistance to facilitate a better understanding of epidemic trends and make attentiveness for scarlet fever control strategies more effective.

## Figures and Tables

**Table 1:** Descriptive information of the outcome and predictor variables.

Variables	Mean	SD	Percentiles		
			25%	Median	75%
Cases	33.00	47.26	4.75	16.00	43.00
PM_2.5_ (µg/m^3^)	91.71	35.32	68.63	82.80	105.7
PM_10_ (µg/m^3^)	121.77	43.40	92.4	113.95	144.27
SO_2_ (µg/m^3^)	28.06	26.12	9.0	18.40	41.10
NO_2_ (µg/m^3^)	52.75	18.99	39.50	51.85	66.57
O_3_ (µg/m^3^)	117.40	55.88	62.62	118.25	169.05
CO (mg/m^3^)	1.73	55.88	1.20	1.50	2.00
MRF (inches)	1.46	1.70	0.10	0.60	2.90
AAP (hPa)	992.11	16.29	982.07	992.10	1004.27
AT (°C)	12.26	10.70	3.90	12.90	21.87
ARH (%)	57.06	11.85	46.10	57.30	68.40
AWS (km/h)	2.10	0.42	1.80	2.00	2.30
ASH (h)	6.54	1.34	5.80	2.80	7.60

AAP: average air pressure; ARH: average relative humidity; AWS: average wind speed; ASH: sunlight hour; AT: average temperature; MRF: monthly rainfall.

**Table 2:** Results of the OLS model, spatial lag model (SLM) and SEM assessing the correlates of scarlet fever with the maximum likelihood estimation.

Variable	Ordinary Least Squares Model				Spatial Lag Model				Spatial Error Model			
	Coefficient	St-Error	T-Stat	*p*-Value	Coefficient	St-Error	Z-Value	*p*-Value	Coefficient	St-Error	Z-Value	*p*-Value
PM_2.5_	0.04012	0.1249	0.3210	0.748	0.04475	0.1224	0.3655	0.715	0.04227	0.1228	0.3441	0.730
PM_10_	0.08048	0.0866	0.9293	0.353	0.09361	0.0849	1.1025	0.270	0.08502	0.0852	0.9974	0.318
SO_2_	0.0004	0.1095	0.0040	0.996	0.00112	0.1073	0.0105	0.992	0.00122	0.1075	0.0113	0.991
NO_2_	0.4514	0.1605	2.8115	0.005	0.35493	0.1609	2.2053	0.027	0.42494	0.1604	2.6485	0.008
O_3_	0.0547	0.0842	0.6499	0.516	0.05085	0.0825	0.6165	0.537	0.05213	0.0827	0.6303	0.528
CO	−7.5136	6.8550	−1.0961	0.273	−7.96663	6.7180	−1.1859	0.235	−7.64643	6.7307	−1.1361	0.255
ARF	5.9287	2.6839	2.2090	0.027	5.49800	2.6347	2.0868	0.036	5.81891	2.6381	2.2057	0.027
AAP	0.0938	0.1490	0.6292	0.529	0.05512	0.1481	0.3722	0.709	0.09062	0.1476	0.6140	0.539
AT	−0.4282	0.6074	−0.7050	0.481	−0.48583	0.5967	−0.8143	0.415	−0.42938	0.5983	−0.7176	0.473
ARH	−0.7329	0.3277	−2.2366	0.025	−0.68165	0.3215	−2.1201	0.034	−0.71913	0.3223	−2.2312	0.025
AWS	−10.0891	6.4000	−1.5764	0.116	−9.69804	6.2731	−1.5460	0.122	−10.01692	6.3059	−1.5885	0.112
ASH	3.9395	1.9747	1.9950	0.047	3.81360	1.9360	1.9698	0.048	3.92472	1.9432	2.0197	0.043
LAMDA (λ)									0.092419	0.3795	0.2435	0.807
Rho (ρ)					0.3616							
R^2^	0.0741				0.0786				0.0743			
LLR	−1819.69				−1819.04				−1819.67			
AIC	3665.38				3665.08				3665.36			

AAP: average air pressure, ARH: average relative humidity, AWS: average wind speed, ASH: sunlight hour, AT: average temperature, MRF: monthly rainfall, LLR: Log Likelihood Ratio, AIC: Akaike Information Criterion, *p* < 0.05 level.

## Supplementary Materials

The following are available online at www.mdpi.com/1660-4601/13/11/1083/s1, Figure S1. Spatial contiguity weights: rooks and queens, Figure S2. Global Moran’s spatial autocorrelation analysis of scarlet fever in Beijing-2013 and 2014. Table S1. Results of tests to determine specific fixed effects and spatial dependency.

## Abbreviations

The following abbreviations are used in this manuscript:

AAP	average atmospheric pressure
ARH	average relative humidity
AT	average temperature
ARH	average relative humidity
MRF	monthly rainfall
ASH	average sunshine hour
AWS	average wind speed
PM	particulate matter
China CDC	China Center for Disease Control and Prevention
GWR	geographically-weighted regression models
LM	Lagrange multiplier
LLR	Log likelihood ratio
Robust LM	robust Lagrange multiplier
AIC	Akaike Information Criterion
