# Supplementary material for: The Association between Environmental Factors and Scarlet Fever Incidence in Beijing Region: Using GIS and Spatial Regression Models
Source: Int J Environ Res Public Health. 2016 Nov 4;13(11):1083. doi: 10.3390/ijerph13111083 (PMC5129293; doi:10.3390/ijerph13111083)
Supplement: Supplementary file 1 [file ijerph-13-01083-s001.pdf]

Supplementary Materials: The Association between Environmental Factors and Scarlet Fever Incidence in Beijing Region: Using GIS and Spatial Regression Models

Gehendra Mahara, Chao Wang, Kun Yang, Sipeng Chen, Jin Guo, Qi Gao, Wei Wang, Quanyi Wang and Xiuhua Guo

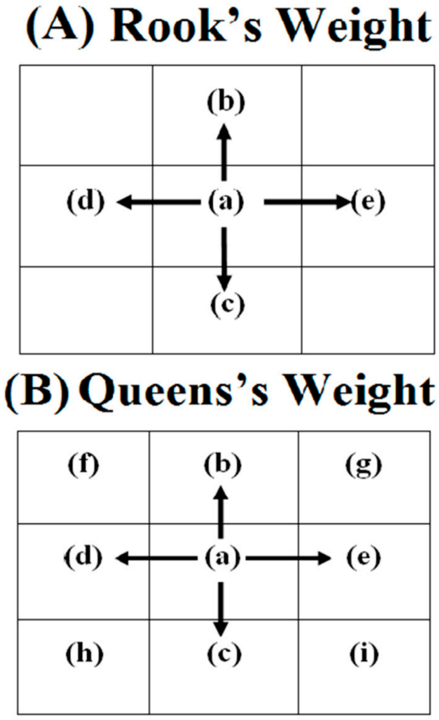

Figure S1. Spatial contiguity weights: Rooks and Queens: (**A**) Rook's Weight; (**B**) Queens's Weight.

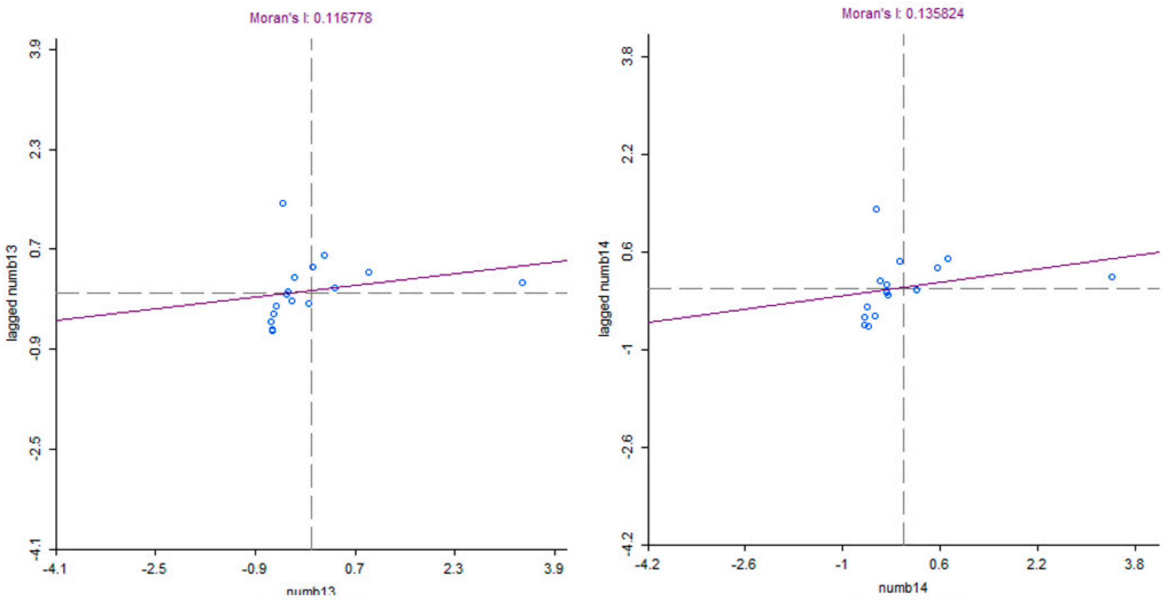

A. Scarlet_13 B. Scarlet_14

Figure S2. Global Moran's Spatial Autocorrelation Analysis of Scarlet Fever in Beijing-2013 and 2014. X-axis standardized for the each geographic object, where the Y-axis represents the mean standardized neighbor value, and points for various locations are based on the "High-high (H-H)/Low-Low (L-H)" classification scheme. Because the data are Z-scores, the raw data have been standardized so that its mean value is zero. (0, 0) is the value of the cross-hairs of the graph. The values in the graph correspond to standard deviations and the slope of the best-fit (regression) line through the points are proportional to the global Moran's *I* for the 6 dataset. The Moran's *I* value and significance can be also found in the log output.

Table S1. Results of tests to determine specific fixed effects and spatial dependency.

Type of Test	Test to Determine the Inclusion of the Model with Spatially Lagged or Spatial Error Auto Correlated Term				
	**LM (SARMA)**	**LM (lag)**	**Robust LM (Lag)**	**LM (Error)**	**Robust LM (Error)**
Statistics Value	7.5708	8.7043	7.5618	0.0089	5.9850
p-Value	0.0027	0.00317	0.00596	0.0002	0.0014

© 2016 by the authors. Submitted for possible open access publication under the terms and conditions of the Creative Commons Attribution (CC-BY) license (http://creativecommons.org/licenses/by/4.0/).
